# Supplementary material for: Overestimated cytotoxicity and underestimated whitening efficacy of glabridin: A result of its poor solubility in DMSO
Source: PLoS One. 2025 Jun 6;20(6):e0325247. doi: 10.1371/journal.pone.0325247 (PMC12143567; doi:10.1371/journal.pone.0325247)

**Supporting Information**

**Overestimated cytotoxicity and underestimated whitening efficacy of glabridin:A result of its poor solubility in DMSO**

Haiyan Liu^1^, Anning Wang^1^, Xiaoyi Chen^3^, Sen Hou^1,2,^* , and Anzhang Li^1,2,^*

^1^Guangzhou Fanzhirong Cosmetics Co. Ltd.

^2^Guangzhou Qingnang Biotechnology Co. Ltd.

^3^Guangzhou Zhongkejian Technology Testing Co., Ltd.

*Corresponding author

Email address: housen@guyu.com.cn, lianzhang@guyu.com.cn (SH, AZL)

**Table of Contents**

Table S1: Original data for the concentration changes of DMSO-dissolved glabridin after dilution shown in Fig 2a.

Table S2: Original data for the concentration changes of CD-encapsulated glabridin after dilution shown in Fig 2a.

Table S3: Original data for the concentration changes of DMSO-dissolved glabridin after dilution shown in Fig 2b.

Table S4: Original data for the concentration changes of CD-encapsulated glabridin after dilution shown in Fig 2b.

Table S5: Original data for the ratio of remained glabridin after dilution shown in Fig 2c.

Table S6: Original data for time dependent cytotoxicity of DMSO-dissolved glabridin shown in Fig 4a, the time span between sample preparation and cell exposure was 0 min.

Table S7: Original data for time dependent cytotoxicity of CD-encapsulated glabridin shown in Fig 4b.

Table S8: Original data for time dependent cytotoxicity of DMSO-dissolved glabridin shown in Fig 4c, the time span between sample preparation and cell exposure was 30 min.

Table S9: Original data for cytotoxicity of CD shown in Fig 4d.

Table S10: Original data for cytotoxicity of CD shown in Fig 4e.

Table S11: Original data for melanin synthesis after DMSO-dissolved glabridin treatment shown in Fig 5a.

Table S12: Original data for melanin synthesis after CD-encapsulated glabridin treatment shown in Fig 5b.

Table S13: Original data for inhibition of melanin shown in Fig 5c.

Fig S1: Glabridin/DMSO solution diluted with water (1:1, v/v).

Table S1: Original data for the concentration changes of DMSO-dissolved glabridin after dilution shown in Fig 2a.

| Original glabridin concentration (µg/mL) | Glabridin concentration after dilution (µg/mL) | | | | |
| --- | --- | --- | --- | --- | --- |
|  | Parallel 1 | Parallel 2 | Parallel 3 | Average | SD |
| 6.25 | ＜1 | ＜1 | ＜1 | ＜1 | 0.0000 |
| 12.5 | ＜1 | ＜1 | ＜1 | ＜1 | 0.0000 |
| 25 | 12.4000 | 13.4290 | 13.4405 | 13.0900 | 0.5971 |
| 50 | 32.4600 | 31.9895 | 31.4729 | 31.9749 | 0.4949 |
| 100 | 67.7700 | 67.3042 | 69.0480 | 68.0411 | 0.9027 |

Table S2: Original data for the concentration changes of CD-encapsulated glabridin after dilution shown in Fig 2a.

| Original glabridin concentration (µg/mL) | Glabridin concentration after dilution (µg/mL) | | | | |
| --- | --- | --- | --- | --- | --- |
|  | Parallel 1 | Parallel 2 | Parallel 3 | Average | SD |
| 6.25 | 5.3358 | 4.6674 | 4.2357 | 4.7463 | 0.5543 |
| 12.5 | 10.4355 | 11.3457 | 12.3598 | 11.3803 | 0.9626 |
| 25 | 24.9803 | 23.9857 | 24.0362 | 24.3341 | 0.5602 |
| 50 | 52.3904 | 52.3069 | 51.2124 | 51.9699 | 0.6573 |
| 100 | 102.2033 | 103.0255 | 102.2169 | 102.4819 | 0.4708 |

Table S3: Original data for the concentration changes of DMSO-dissolved glabridin after dilution shown in Fig 2b.

| Incubation time after dilution (min) | Glabridin concentration after dilution (µg/mL) | | | | |
| --- | --- | --- | --- | --- | --- |
|  | Parallel 1 | Parallel 2 | Parallel 3 | Average | SD |
| 0 | 65.4686 | 65.1491 | 66.0272 | 65.5483 | 0.4444 |
| 5 | 62.496 | 63.468 | 62.1222 | 62.6954 | 0.6947 |
| 10 | 52.4052 | 53.1157 | 50.4594 | 51.9934 | 1.3752 |
| 15 | 31.5843 | 30.906 | 32.3544 | 31.6149 | 0.7247 |
| 30 | 5.46037 | 5.2159 | 6.07488 | 5.5837 | 0.4426 |
| 60 | ＜1 | ＜1 | ＜1 | ＜1 | 0.0000 |
| 120 | ＜1 | ＜1 | ＜1 | ＜1 | 0.0000 |

Table S4: Original data for the concentration changes of CD-encapsulated glabridin after dilution shown in Fig 2b.

| Incubation time after dilution (min) | Glabridin concentration after dilution (µg/mL) | | | | |
| --- | --- | --- | --- | --- | --- |
|  | Parallel 1 | Parallel 2 | Parallel 3 | Average | SD |
| 0 | 103.0003 | 104.7047 | 104.7023 | 104.1358 | 0.9833 |
| 5 | 103.675 | 102.8175 | 102.0601 | 102.8509 | 0.8080 |
| 10 | 102.5878 | 103.4735 | 102.9458 | 103.0024 | 0.4456 |
| 15 | 105.5302 | 105.6085 | 105.8152 | 105.6513 | 0.1472 |
| 30 | 100.6302 | 100.8875 | 101.442 | 100.9866 | 0.4149 |
| 60 | 104.263 | 103.6705 | 103.4566 | 103.7967 | 0.4178 |
| 120 | 102.4576 | 102.5624 | 103.2575 | 102.7592 | 0.4347 |

Table S5: Original data for the ratio of remained glabridin after dilution shown in Fig 2c.

| Original glabridin concentration (µg/mL) | DMSO-dissolved glabridin | | CD-encapsulated glabridin | |
| --- | --- | --- | --- | --- |
|  | Remained glabridin in solution (%) | SD | Remained glabridin in solution (%) | SD |
| 6.25 | 0.0000 | 0.0000 | 75.9408 | 8.8684 |
| 12.50 | 0.0000 | 0.0000 | 91.0427 | 7.7009 |
| 25.00 | 52.3601 | 2.3884 | 97.3363 | 2.2409 |
| 50.00 | 63.9498 | 0.9897 | 103.9398 | 1.3147 |
| 100.00 | 68.0411 | 0.9027 | 102.4819 | 0.4708 |

Table S6: Original data for time dependent cytotoxicity of DMSO-dissolved glabridin shown in Fig 4a, the time span between sample preparation and cell exposure was 0 min.

| Original glabridin concentration (µg/mL) | Cytotoxicity (%) | | | | |
| --- | --- | --- | --- | --- | --- |
|  | Parallel 1 | Parallel 2 | Parallel 3 | Average | SD |
| 0.78 | 93.79 | 81.67 | 89.88 | 88.45 | 6.18 |
| 1.56 | 99.05 | 87.34 | 82.08 | 89.49 | 8.69 |
| 3.13 | 88.87 | 86.23 | 87.39 | 87.49 | 1.32 |
| 6.25 | 78.81 | 80.53 | 84.08 | 81.14 | 2.68 |
| 12.50 | 68.99 | 69.42 | 78.94 | 72.45 | 5.62 |
| 25.00 | 40.76 | 38.48 | 30.36 | 36.53 | 5.47 |
| 50.00 | 14.42 | 14.40 | 14.20 | 14.34 | 0.12 |
| 100.00 | 14.03 | 13.97 | 14.16 | 14.05 | 0.10 |

Table S7: Original data for time dependent cytotoxicity of CD-encapsulated glabridin shown in Fig 4b.

| Original glabridin concentration (µg/mL) | Cytotoxicity (%) | | | | |
| --- | --- | --- | --- | --- | --- |
|  | Parallel 1 | Parallel 2 | Parallel 3 | Average | SD |
| 0.78 | 98.09 | 98.87 | 99.81 | 98.92 | 0.86 |
| 1.56 | 100.83 | 97.94 | 97.78 | 98.85 | 1.71 |
| 3.13 | 96.84 | 98.58 | 98.70 | 98.04 | 1.04 |
| 6.25 | 95.43 | 94.82 | 94.63 | 94.96 | 0.42 |
| 12.50 | 76.53 | 79.11 | 79.88 | 78.50 | 1.76 |
| 25.00 | 62.00 | 63.01 | 63.32 | 62.78 | 0.69 |
| 50.00 | 25.65 | 25.97 | 25.37 | 25.66 | 0.30 |
| 100.00 | 5.48 | 5.08 | 5.52 | 5.36 | 0.24 |

Table S8: Original data for time dependent cytotoxicity of DMSO-dissolved glabridin shown in Fig 4c, the time span between sample preparation and cell exposure was 30 min.

| Original glabridin concentration (µg/mL) | Cytotoxicity (%) | | | | |
| --- | --- | --- | --- | --- | --- |
|  | Parallel 1 | Parallel 2 | Parallel 3 | Average | SD |
| 156.25 | 117.49 | 110.36 | 114.40 | 114.08 | 3.58 |
| 312.50 | 109.96 | 112.48 | 110.36 | 110.93 | 1.35 |
| 625.00 | 101.27 | 105.97 | 105.33 | 104.19 | 2.55 |
| 1250.00 | 104.91 | 102.75 | 102.45 | 103.37 | 1.34 |
| 2500.00 | 94.80 | 94.98 | 94.56 | 94.78 | 0.21 |
| 5000.00 | 92.96 | 92.23 | 91.65 | 92.28 | 0.66 |
| 10000.00 | 87.68 | 88.75 | 86.85 | 87.76 | 0.95 |
| 20000.00 | 77.99 | 78.56 | 79.43 | 78.66 | 0.73 |

Table S9: Original data for cytotoxicity of CD shown in Fig 4d.

| CD (µg/mL) | Cytotoxicity (%) | | | | |
| --- | --- | --- | --- | --- | --- |
|  | Parallel 1 | Parallel 2 | Parallel 3 | Average | SD |
| 0 | 100.29 | 100.27 | 99.42 | 99.99 | 0.50 |
| 72.48 | 102.42 | 102.10 | 99.32 | 101.28 | 1.71 |
| 144.96 | 99.19 | 98.55 | 100.11 | 99.28 | 0.79 |
| 289.92 | 95.53 | 101.07 | 96.43 | 97.68 | 2.97 |
| 579.84 | 100.15 | 94.02 | 99.22 | 97.80 | 3.30 |
| 1159.69 | 93.61 | 97.53 | 95.56 | 95.57 | 1.96 |
| 2319.38 | 96.41 | 97.99 | 96.33 | 96.91 | 0.94 |
| 4638.75 | 93.99 | 99.09 | 93.30 | 95.46 | 3.16 |
| 9277.50 | 90.59 | 84.26 | 85.78 | 86.88 | 3.30 |

Table S10: Original data for cytotoxicity of DMSO shown in Fig 4e.

| DMSO (µg/mL) | Cytotoxicity (%) | | | | |
| --- | --- | --- | --- | --- | --- |
|  | Parallel 1 | Parallel 2 | Parallel 3 | Average | SD |
| 0 | 99.35 | 101.53 | 99.99 | 100.29 | 1.12 |
| 1000.00 | 98.28 | 100.84 | 98.91 | 99.34 | 1.33 |
| 5000.00 | 97.03 | 98.63 | 98.85 | 98.17 | 0.99 |
| 10000.00 | 87.91 | 92.72 | 90.72 | 90.45 | 2.42 |
| 25000.00 | 48.36 | 48.55 | 48.36 | 48.42 | 0.11 |
| 50000.00 | 44.80 | 45.42 | 45.80 | 45.34 | 0.50 |
| 100000.00 | 10.25 | 10.50 | 10.68 | 10.48 | 0.22 |
| 150000.00 | 4.06 | 4.12 | 4.12 | 4.10 | 0.03 |
| 200000.00 | 3.94 | 3.87 | 3.87 | 3.89 | 0.04 |

Table S11: Original data for melanin synthesis after DMSO-dissolved glabridin treatment shown in Fig 5a.

| Original glabridin concentration (µg/mL) | Absorbance at 405 nm | | | | |
| --- | --- | --- | --- | --- | --- |
|  | Parallel 1 | Parallel 2 | Parallel 3 | Average | SD |
| 0.78 | 0.1309 | 0.1341 | 0.1321 | 0.1324 | 0.0016 |
| 3.125 | 0.1295 | 0.1231 | 0.1231 | 0.1252 | 0.0037 |
| NC | 0.1628 | 0.1688 | 0.1616 | 0.1644 | 0.0039 |
| BC | 0.0505 | 0.0501 | 0.0516 | 0.0507 | 0.0008 |

Table S12: Original data for melanin synthesis after CD-encapsulated glabridin treatment shown in Fig 5b.

| Original glabridin concentration (µg/mL) | Absorbance at 405 nm | | | | |
| --- | --- | --- | --- | --- | --- |
|  | Parallel 1 | Parallel 2 | Parallel 3 | Average | SD |
| 0.78 | 0.1132 | 0.1122 | 0.1102 | 0.1119 | 0.0015 |
| 3.125 | 0.1009 | 0.1063 | 0.1045 | 0.1039 | 0.0027 |
| NC | 0.1386 | 0.1402 | 0.1334 | 0.1374 | 0.0036 |
| BC | 0.0585 | 0.0581 | 0.0576 | 0.0581 | 0.0005 |

Table S13: Original data for inhibition of melanin shown in Fig 5c.

| Original glabridin concentration (µg/mL) | DMSO-dissolved glabridin | | CD-encapsulated glabridin | |
| --- | --- | --- | --- | --- |
|  | Melanin inhibition rate (%) | SD | Melanin inhibition rate (%) | SD |
| 0.78 | 28.17 | 1.16 | 32.18 | 1.57 |
| 3.125 | 34.45 | 2.67 | 42.23 | 2.83 |

Fig S1: Glabridin/DMSO solution diluted with water (1:1, v/v).


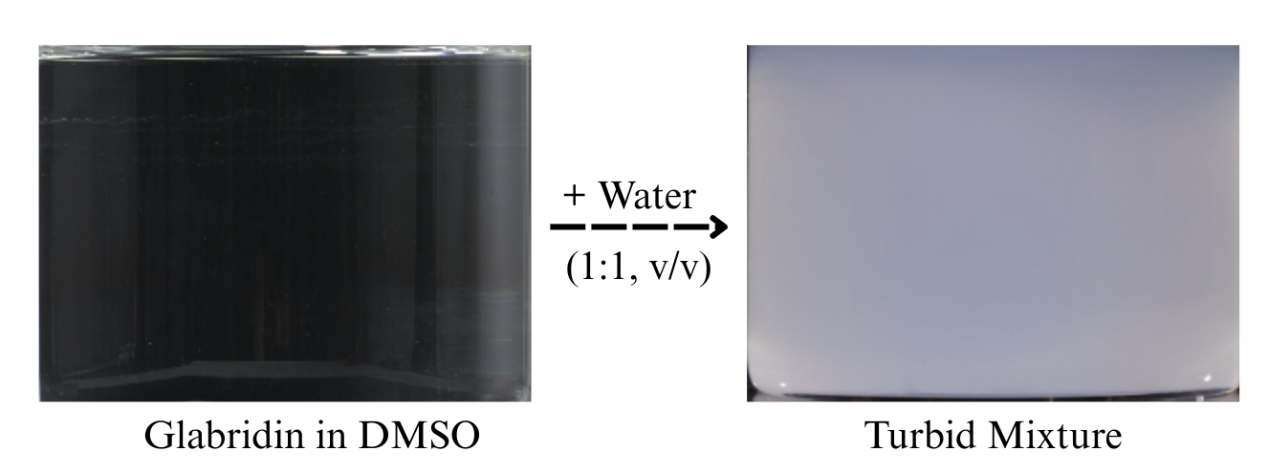

Supplement: S1 File — (DOCX) [file pone.0325247.s001.docx]
