# Supplementary material for: Overestimated cytotoxicity and underestimated whitening efficacy of glabridin: A result of its poor solubility in DMSO
Source: PLoS One. 2025 Jun 6;20(6):e0325247. doi: 10.1371/journal.pone.0325247 (PMC12143567; doi:10.1371/journal.pone.0325247)
Supplement: Attachment — Submitted filename: Response to Reviewers.doc [file pone.0325247.s003.doc]

Dear Editor：

Thanks a million for your effort for sending our paper for reviewing. We are delighted by the reviewers’ positive comments, such as “**This is a significant contribution to the field of cosmetic science**” and “**I recommend its publication**”. We have revised the manuscript according to the reviewers’ comments and the editor’s suggestions. We have answered the concerns about the grants and showed the funding did not influence the scientific quality of the paper. We have supplemented additional experiments and improved the language. We have changed the figures according to the comments. We have supplemented additional discussion to address the concerns of the reviewers. We believe that this version of manuscript has been greatly improved due to the constructive comments of reviewers.

The changes have been highlighted in the manuscript. Please see the point-by-point response as follows:

**Editors**

**1. Comment:** *Please ensure that your manuscript meets PLOS ONE's style requirements, including those for file naming.*

**Response:** We have revised the manuscript according to style requirements.

**Changes:** In the corresponding author section (lines 11-12) the name of the corresponding author has been removed, and the corresponding author’s initials (SH, AZL) has been added.

**2. Comment:** *Thank you for stating the following financial disclosure: This study was supported by a scientific grant from Guangzhou Fanzhirong Cosmetics Co., Ltd (MT-2023-002). Please state what role the funders took in the study. If the funders had no role, please state: "The funders had no role in study design, data collection and analysis, decision to publish, or preparation of the manuscript." If this statement is not correct you must amend it as needed. Please include this amended Role of Funder statement in your cover letter; we will change the online submission form on your behalf.*

**Response:** According to the suggestion, in the cover letter we added “Role of Funder Statement”, confirming that the funder had no involvement in any aspect of this study.

**Changes:** In the Acknowledgments section, all funding-related descriptions have been completely removed. The following statement has been added in the cover letter: "This study was supported by a scientific grant from Guangzhou Fanzhirong Cosmetics Co., Ltd (MT-2023-002). The funder had no role in study design, data collection and analysis, decision to publish, or preparation of the manuscript."

**3. Comment:** *Thank you for stating the following in the Acknowledgments Section of your manuscript: This study was supported by a scientific grant from Guangzhou Fanzhirong Cosmetics Co., Ltd. (MT-2023-002).We note that you have provided funding information that is not currently declared in your Funding Statement. However, funding information should not appear in the Acknowledgments section or other areas of your manuscript. We will only publish funding information present in the Funding Statement section of the online submission form. 
Please remove any funding-related text from the manuscript and let us know how you would like to update your Funding Statement. Currently, your Funding Statement reads as follows: 
This study was supported by a scientific grant from Guangzhou Fanzhirong Cosmetics Co., Ltd (MT-2023-002).Please include your amended statements within your cover letter; we will change the online submission form on your behalf.*

**Response:** According to the suggestion, we have deleted the funding-related text from the manuscript and added “Role of Funder Statement” in the cover letter.

**Changes:** In the Acknowledgments section, all funding-related descriptions have been completely removed. In the cover letter Section add "This study was supported by a scientific grant from Guangzhou Fanzhirong Cosmetics Co., Ltd. (MT-2023-002).The funder had no role in study design, data collection and analysis, decision to publish, or preparation of the manuscript. " We also supplemented the funding information in the Funding Statement section of the online submission form.

**4. Comment:** *We note that you have included the phrase "data not shown" in your manuscript. Unfortunately, this does not meet our data sharing requirements. PLOS does not permit references to inaccessible data. We require that authors provide all relevant data within the paper, Supporting Information files, or in an acceptable, public repository. Please add a citation to support this phrase or upload the data that corresponds with these findings to a stable repository (such as Figshare or Dryad) and provide and URLs, DOIs, or accession numbers that may be used to access these data. Or, if the data are not a core part of the research being presented in your study, we ask that you remove the phrase that refers to these data.*

**Response:** According to the suggestion, we have added the data in the supporting information as Fig S1.

**Changes:** In lines 200-201, change “data not shown” to “Fig S1 in supporting information” .

Add Fig S1 in supporting information.

**Reviewer 1**

**1. Comment:** *The study optimizes glabridin use in skin whitening applications by using CD-encapsulated glabridin, which reduces variation and improves water solubility, stability, and bioactivity. This provides insights for future studies and lays a foundation for safer, more effective whitening products.* ***It is a good effort*** *to find complex information of science required to correctly evaluate the cytotoxicity and effectiveness.*

**Response:** We are delighted by the reviewer's positive comments concerning our paper.

**Reviewer 2**

**1. Comment:** *The manuscript is well written and contains some important information therefore* ***i recommend its publication****. However authors need to carefully revise the whole manuscript to avoid any grammatical and spells mistake.*

**Response:** We are delighted by the reviewer's positive comments. According to the reviewer's suggestion, we have corrected grammatical and spells mistakes.

**Reviewer 3**

**1. Comment:** *The manuscript describes a comparative study that addresses a crucial concern related to safe-usage of glabridin, used as a whitening agent. Importantly, authors have evaluated cytotoxicity mediated by glabridin, if dissolved DMSO. Further, as an alternative to DMSO, authors showed that cyclodextrin (CD) mediated encapsulation, serve as better solvent with reduced cytotoxity and increased whitening efficiency. However, it should also be taken into account that the importance of cyclodextrin in terms of solubility and bioavailability is already studied.*

**Response:** The purpose of our study is to show that in previous studies that used DMSO as solvent for glabridin, the cytotoxicity of glabridin was overestimated. CD was simply used as a control to show that if the solubility problem of DMSO was avoided by using CD instead, the overestimated cytotoxicity would not exist. Herein the reported solubility and bioavailability of CD does not hamper the originality of our study. We have added a discussion to clarify our point of view.

**Changes:** In Lines 293-298 add "Although previous studies have well established cyclodextrin's role in enhancing the bioavailability of poorly soluble compound [10], our work still makes a key advance beyond existing bioavailability researches. In this study, CD was simply used as a control to show that if the solubility problem of DMSO was avoided by using CD instead, the overestimated cytotoxicity would not exist. Herein, the cytotoxicity of glabridin might be overestimated by previous studies using DMSO as the solvent."

**2. Comment:** *Statistical significance is not shown in most of the data. Kindly provide all the data with proper significance.*

**Response:** According the suggestion, we have supplemented statistical significance in our data presentation in Fig 2, Fig 4 and Fig 5.

**Changes:** We have replaced Fig 2, Fig 4 and Fig 5 with new images with additional statistical significance information. We have also correct corresponding text in figure captions of Fig 2, Fig 4 and Fig 5 and in Materials and Methods section.

**3. Comment:***Line 168-169: Authors mentioned that "glabridin enter the hydrophobic cavity of CD, getting trapped within the CD molecule" Please mention the reference for the mechanism of CD mediated solubility of glabridin.*

**Response:** According to the suggestion, we have added a new reference and provide a discussion to explain the mechanism.

**Changes:** In lines 175-180, add "Previous studies have demonstrated that glabridin forms a 1:1 stoichiometric inclusion complex with CD through characteristic host-guest interactions, where the hydrophobic C-ring of glabridin enters the cyclodextrin cavity [10]. This interaction is driven by hydrophobic interactions between the nonpolar cavity and glabridin's lipophilic structure. Meanwhile, the hydrophilic exterior of CD enhances aqueous solubility."

**4.** **Comment:** *Line 169: Authors mentioned that “HPLC revealed that the encapsulation rate of glabridin in CD was 9.73%”. Please provide the reference for the statement.*

**Response:** According to the suggestion, we have supplemented additional reference and detailed the experimental protocol.

**Changes:** In lines 92-95 add "The drug loading efficiency (DLE) was determined by Eq 1:

*DLE =* *×* 100%	(1)

where *C*_0_ represents the total mass of CD-glabridin complex, and *C*_1_ represents the mass of glabridin loaded within the complex, as determined by HPLC analysis of glabridin content."

In line 107, add reference [19] to support HPLC analysis.

**5. Comment:** *Line 172: Authors mentioned that “maximum glabridin concentration typically used in cytotoxicity assays is less than 100 µg/mL”. It requires appropriate reference.*

**Response:** According to the suggestion, we have supplemented three additional references where the concentrations of glabridin in the experiments are below 100 µg/mL.

**Changes:** In line 182, add three new references [9], [12] and [17].

**6. Comment:** *Figure 1: Need to provide the reference for the diagram showing CD-mediated encapsulation of glabridin.*

**Response:** According to the suggestion, we have added a new reference and provide a discussion to explain the mechanism.

**Changes:** In lines 175-180, add "Previous studies have demonstrated that glabridin forms a 1:1 stoichiometric inclusion complex with CD through characteristic host-guest interactions, where the hydrophobic C-ring of glabridin enters the cyclodextrin cavity [10]. This interaction is driven by hydrophobic interactions between the nonpolar cavity and glabridin's lipophilic structure. Meanwhile, the hydrophilic exterior of CD enhances aqueous solubility."

**7. Comment:** *Line 193: Need to correct the figure reference in the text from 2c to 2b.*

**Response:** According to the suggestion, we have corrected the figure reference.

**Changes:** In Line 214 change "Fig 2c"to "Fig 2b".

**8. Comment:** *Figure 3: The turbidity images and microscopy images for precipitation and crystals are blurred. Authors should provide high quality and clear images for the same.*

**Response:** According to the suggestion, we have provided high quality and clear images for Fig 3.

**Changes:** In Fig 3, we used a new image with high quality to replace the old one, where the precipitation process and the shape of crystals can be clearly observed.

**9. Comment:** *Line 219-222: Authors speculated two consequences for decreased bioavailability. However, these speculations either should be backed up by some data or must have appropriate references. Same also goes for line 240-252, author should provide data or reference to support physical damage by crystals and their transport.*

**Response**: According to the suggestion, we have supplemented a discussion with supporting literature references to support our point of view.

**Changes:** In lines 246-252 add " Previous studies showed that the shape and aggregation state of calcium oxalate monohydrate (COM) crystals were crucial factors in their toxicity to renal epithelial cells [16]. Crystals with sharp edges caused more severe cell damage, including membrane rupture, increased ROS production, and mitochondrial dysfunction, leading to necrosis. Aggregation of sharp-edged crystals exacerbated cell injury, while aggregation of blunt-edged crystals showed reduced toxicity. These findings pointed out the significant role of crystal shape and aggregation in cytotoxicity. "

**10. Comment:** *Figure 4a, 4b and 4c: Some of error bars shown in these figures does not coincides with graphs. It is unlikely that errors bars get separated from main graph, if generated using trusted scientific software/tools such as Graphpad Prism. Authors should provide images with better quality.*

**Response:** The curves in these figures is the fitting curves rather than data point connections. Therefore, data dots are not located on the curves. We have clarified this point by revising the figure caption so the readers can easily understand it.

**Changes:** In the figure caption of Fig 4 "The curves in a-c are fitting curves for corresponding IC50 calculation."

**11. Comment:** *Figure 4: Cell viability assay should also include controls i.e. effect of DMSO and cyclodextrin alone. This is important to ensure that the observed cell viability is not contributed by the solvents (DMSO or CD) itself.*

**Response:** According to the suggestion, we have supplemented additional experiments concerning cell viability assay of DMSO alone. The data of cell viability assay of CD alone was shown in Fig 4d. The results showed that neither DMSO nor CD alone significantly affected cell viability in the tested concentrations, ensuring that the observed effects are primarily due to glabridin. A discussion was added.

**Changes:** In lines 147-148 add "The concentrations of DMSO were 1000, 5000, 10000, 25000, 50000, 100000, 150000, and 200000 μg/mL, respectively."

Replace Fig 4 with a new image showing cell viability assay of DMSO and alone. The figure caption was also revised accordingly.

In line 265-267 add "Our result well agreed with the literature. Our data showed that DMSO of a concentration below 0.5% did not affect cell viability (Fig 4e)."

**12. Comment:** *Please provide the reference for Melanin synthesis inhibition assay OR author should provide data to validate the usability of the assay. Also provide the protocol for measuring melanin synthesis "rate"*

**Response:** According to the suggestion, we have supplemented a new reference for the melanin synthesis inhibition assay. The protocol was shown in Materials and Methods section “Melanin synthesis inhibition assay”. We also changed the phrase “melanin synthesis rate” to “inhibition of melanin”.

**Changes:** In lines 158. add “The inhibition of melanin was measured with a method developed from a previous literature [20].”

Replace Fig 5 with a new image.

**13. Comment:** *Figure 5: Authors should provide detailed figure legend with the abbreviations (NC and BC etc.) used in the figures. It is difficult to interpret data without it, as it is also not mentioned anywhere in the text.*

**Response:** According to the suggestion, we have added detailed information about the abbreviations *NC* and *BC* in the figure legend and Materials and Methods section.

**Changes:** In the figure legend of Fig 5 add “(BC: blank control; NC: negtive control)”.

In lines 151-156 add “The cells were cultured for 24 h in RPMI 1640 Medium and then the medium was replaced with DMEM containing glabridin at different concentrations. After cell culture with these glabridin solutions for 24 h, the culture medium was removed and the cells were lysed using 1 mol/L NaOH containing 10% DMSO. The mixtures were incubated at 80 ℃ for 1 h and then transferred to 96-well plates. Cells cultured in RPMI 1640 medium were used as blank control (BC). Cells treated with DMEM without glabridin was used as negative control (NC). ”

**Reviewer 4**

**1. Comment:** *Include more quantitative data on crystal formation (e.g., size distribution, morphology) using microscopy or other imaging techniques.*

**Response:** According to the suggestion, we have supplemented microscopy images with high resolution in Fig 3. The morphology and structural details of the crystals can be clearly observed.

**Changes:** Replace Fig 3 with a new image where the morphology and structural details of the crystals can be clearly observed.

**2. Comment:** *Explore additional solvents or formulations beyond CD encapsulation to provide a broader solution framework.*

**Response:** According to the suggestion, we have supplemented a discussion concerning a broader alternative solvents and formulation strategies beyond cyclodextrin (CD) encapsulation.

**Changes:** In lines 185-193 add "Various formulation strategies have also been applied to improve the solubility and physical stability of poorly soluble ingredients, such as glabridin. For instance, both liposome-based delivery systems and nanoemulsions have been reported to effectively prevent the precipitation of glabridin [22,23]. These systems not only enhance the dispersion of glabridin in aqueous environment but also improve its stability. However, these approaches still suffer from certain shortcomings. Nanoemulsions typically use surfactants, which would introduce potential skin irritation [24]. Liposome strategy requires complicated equipment, such as high-pressure homogenizer, and likely introduces additional cytotoxicity. CD encapsulation provides a simpler and more robust approach with favorable formulation compatibility."

**3. Comment:** *Conduct in vivo studies to validate findings under conditions that mimic real-world cosmetic applications.*

**Response:** The aim of our study was to point out an overestimation in cytotoxicity of glabridin that occurs in cell experiments in previous studies. We did not think such overestimation also happened *in vivo* experiments. The overestimated cytotoxicity caused by precipitation was a result of the dilution process. In cell experiment, dilution of glabridin was inevitable. However, *in vivo* experiment, glabridin does not experience dilution process. Therefore, the overestimated cytotoxicity is unlikely to occur in *in vivo* studies.

To clarify this point of view, a discussion was added in the Discusion section.

**Changes:** In lines 362-368 add "The aim of our study was to highlight the overestimated cytotoxicity and underestimated whitening efficacy of glabridin observed in cell-based experiments. We do not believe that such overestimation and underestimation occur in *in vivo* studies. These effects were caused by precipitation resulting from the dilution process. In *in vitro* experiments, dilution of glabridin is inevitable. However, in *in vivo* conditions, glabridin does not undergo the same dilution process. Therefore, the overestimated cytotoxicity and underestimated whitening efficacy are unlikely to occur in *in vivo* studies. "

**4. Comment:** *Address potential biases by encouraging independent replication or collaboration with academic institutions.*

**Response:** Our study does not contain any bias. All data were measured scientifically and precisely by at least three repetitions. We declared that the grant funder had no role in study design, data collection and analysis, decision to publish, or preparation of the manuscript.

**Changes:** In the cover letter Section add "This study was supported by a scientific grant from Guangzhou Fanzhirong Cosmetics Co., Ltd. (MT-2023-002).The funder had no role in study design, data collection and analysis, decision to publish, or preparation of the manuscript. "

**5. Comment:** *Expand on methodological details, particularly regarding CD encapsulation protocols and comparative analysis techniques.*

**Response:** According to the suggestion, we have expanded on the methodological details related to CD encapsulation in the revised manuscript.

**Changes:** In lines 92-95 add "The drug loading efficiency (DLE) was determined by Eq 1:

*DLE =* *×* 100%	(1)

where *C*_0_ represents the total mass of CD-glabridin complex, and *C*_1_ represents the mass of glabridin loaded within the complex, as determined by HPLC analysis of glabridin content."

In line 107, add reference [19] to support HPLC analysis.

**6. Comment:** *Provide a more balanced discussion by acknowledging limitations in generalizing findings across all prior studies.*

**Response:** According to the suggestion, we have added a discussion to acknowledging limitations in generalizing findings across previous studies. Specifically, we limited our findings in vitro cell experiment. We pointed out in vivo experiments do not experience the same experimental conditions so that the conclusion of our study is probably not valid for in vivo studies. We also discussed other strategies to improve the solubility of glabridin.

**Changes:** In lines 185-193 add " Various formulation strategies have also been applied to improve the solubility and physical stability of poorly soluble ingredients, such as glabridin. For instance, both liposome-based delivery systems and nanoemulsions have been reported to effectively prevent the precipitation of glabridin [22,23]. These systems not only enhance the dispersion of glabridin in aqueous environment but also improve its stability. However, these approaches still suffer from certain shortcomings. Nanoemulsions typically use surfactants, which would introduce potential skin irritation [24]. Liposome strategy requires complicated equipment, such as high-pressure homogenizer, and likely introduces additional cytotoxicity. CD encapsulation provides a simpler and more robust approach with favorable formulation compatibility. "

In lines 362-368 add "The aim of our study was to highlight the overestimated cytotoxicity and underestimated whitening efficacy of glabridin observed in cell-based experiments. We do not believe that such overestimation and underestimation occur in *in vivo* studies. These effects were caused by precipitation resulting from the dilution process. In *in vitro* experiments, dilution of glabridin is inevitable. However, in *in vivo* conditions, glabridin does not undergo the same dilution process. Therefore, the overestimated cytotoxicity and underestimated whitening efficacy are unlikely to occur in *in vivo* studies. "
